# Associations of the T329S Polymorphism in Flavin-Containing Monooxygenase 3 With Atherosclerosis and Fatty Liver Syndrome in 90-Week-Old Hens

**DOI:** 10.3389/fvets.2022.868602

**Published:** 2022-03-30

**Authors:** Jianlou Song, Xuefeng Shi, Xianyu Li, Qianni Liang, Lingsen Zeng, Guangqi Li, Yiyuan Yan, Guiyun Xu, Jiangxia Zheng

**Affiliations:** ^1^Key Laboratory of Animal Genetics, Breeding and Reproduction of the Ministry of Agriculture, College of Animal Science and Technology, China Agricultural University, Beijing, China; ^2^Beijing Huadu Yukou Poultry Industry Co. Ltd., Beijing, China

**Keywords:** flavin-containing monooxygenase 3, atherosclerosis, fatty liver syndrome, adiposity, old hen

## Abstract

This study aimed to evaluate the effects of the spontaneous genetic mutation T329S in flavin-containing monooxygenase 3 (FMO3) on atherosclerosis (AS), fatty liver syndrome (FLS), and adiposity in 90-week-old layers. At 90 weeks of age, 27 *FMO3* genotyped Rhode Island White chickens (consisting of nine AA hens, nine AT hens, and nine TT hens) with normal laying performance were selected. The AS lesions, incidence of FLS, fat deposition, metabolic characteristics, and production performance of these egg-layers with different *FMO3* genotypes were assessed. The T329S mutation in TT hens reduced the AS lesions (*P* < 0.01) and altered the plasma metabolic indices more than it did in the AA and AT hens. Furthermore, it reduced the incidence of FLS, hepatic triglyceride deposition (*P* < 0.05), liver indices (*P* < 0.05), and fat deposition (*P* < 0.05) in the subcutis and abdomen of TT hens compared to those of AA and AT hens. Moreover, as an effect of T329S, TT hens laid a higher than average number of eggs and maintained a higher egg-laying rate from 68 to 90 weeks than AA and AT hens. Our study confirmed that the T329S mutation in *FMO3* could reduce the development of AS lesions, the incidence of FLS, and fat deposition, which are associated with changes in plasma and hepatic metabolic indices and improvements in the laying performance of older layers. Our results may provide a new strategy for using the T329S mutation to improve the health status and production performance of layers during the late laying period.

## Introduction

Flavin-containing monooxygenases (FMOs; EC 1.14.13.8) are an important class of microsomal enzymes because they can catalyze the oxygenation of soft nucleophilic heteroatom-containing (e.g., nitrogen, sulfur, and phosphorus) organic substances and convert them to more readily excreted polar metabolites. Therefore, FMOs have a significant role in the metabolism and detoxification of pharmaceuticals, endogenous substances, and dietary-derived compounds (1). FMO3 is the most important member of the FMO family. It has key roles in endogenous trimethylamine (TMA) metabolic pathways and can oxidize TMA into trimethylamine N-oxide (TMAO), which is closely linked to many metabolic characteristics (2, 3). For example, several mutations in the *FMO3* gene in humans can inhibit the activity of the FMO3 enzyme during the oxidization of TMA, a substance with a fishy odor, resulting in trimethylaminuria (4). Similarly, chickens with a threonine-to-serine substitution at position 329 (T329S; *FMO3* c.984 A>T) of *FMO3*, which has a function similar to that in humans, is associated with eggs with a fishy odor when chickens are fed a diet with a high-level TMA precursor (e.g., choline, carnitine, betaine) (5, 6). Therefore, the association of *FMO3* with other metabolic diseases involving the TMA metabolic pathway has received much attention.

Recently, a new role of the TMA metabolic pathway in lipid metabolic diseases in mammals has been identified (7). TMAO has been suggested to be a risk factor for atherosclerosis (AS), and TMAO supplementation experiments have demonstrated the role of TMAO in promoting the development of AS and thrombosis in mice (7, 8). An increase in platelet reactivity introduced by cholesterol accumulation in macrophages and the subsequent activation of inflammatory pathways have been considered possible mechanisms involved in the proatherogenic effect of TMAO (7–9). Hepatic FMO3, a TMAO-generating enzyme, was initially identified as a therapeutic target for AS because antisense oligonucleotide (ASO)-mediated silencing of FMO3 decreased TMAO levels and AS lesions in mouse models (10). Furthermore, several researchers have suggested that FMO3 itself is a central regulator of lipid and glucose (Glu) metabolism because *FMO3* ASO decreased plasma cholesterol and Glu levels. They argued that pharmacological inhibition of *FMO3* to reduce TMAO levels would be confounded by metabolic interactions (11, 12). However, more recently, an experiment involving *FMO3* knockout (KO) via CRISPR/Cas9 technology in mice clarified that previous observations of reductions in AS after ASO treatment may have been attributable to the off-target effect on plasma cholesterol levels rather than TMAO levels. Furthermore, it was confirmed that *FMO3* KO reduced systemic TMAO levels and thrombosis potential; however, it had only a minor effect on plasma lipid levels without reduced AS lesions in mice (13). There is a certain association between the TMA metabolic pathway and AS; however, the studies of this pathway have been based on the biotechnological interventions of *FMO3* in mammals, and it is difficult to exclude the off-target effects or artifacts of these biotechnologies (14). To our knowledge, no spontaneous mutation in *FMO3* in relation to AS in animal models has been reported; therefore, the relationship between *FMO3* and AS requires further clarification.

T329S is a spontaneous recessive mutation in *FMO3* that does not require external intervention. It has been identified within a highly conserved FATGY motif, which can change FATGY into FASGY and thus decrease the substrate affinity of FMO3 enzyme. It has been reported is highly associated with TMA metabolic pathways in chickens (5). Previous studies have shown that T329S can diminish the ability of FMO3 to oxidize TMA to TMAO, resulting in a decrease in circulating TMAO concentrations (15, 16). Subsequently, Guo (17) confirmed this claim by showing FMO3 enzyme activity in TT hens (T329S homozygous mutants) was significantly lower than those of AA (wild-type) and AT (heterozygous mutants) hens. Because the regulation of cholesterol disposal in poultry is highly similar to that in mammals, laying hens could be used as an animal model to further explore the relationship between FMO3 and AS (18, 19). Additionally, it is generally accepted that AS, fatty liver syndrome (FLS), and adiposity are closely linked in animals, including layers (20–22). These conditions negatively impact the poultry industry because they reduce the production performance of laying hens during the late laying period (23, 24). In particular, FLS has the most serious impact on egg production performance among these conditions because the liver has an important role in regulating hepatic lipid metabolism and yolk precursor synthesis in layers (25, 26). However, the effects of the T329S mutation on FLS and adiposity of layers remain unknown.

This study aimed to validate the effects of the T329S mutation in *FMO3* on AS lesions in laying hens. Furthermore, it aimed to explore whether the T329S mutation is associated with other lipid metabolic conditions, such as FLS and adiposity, which have not yet been reported. We hypothesized that the TMA metabolic pathway involved in the T329S mutation may have a positive effect on lipid metabolic conditions of 90-week-old layers. The generated data are useful for improving our understanding of the association of the T329S polymorphism in FMO3 with lipid metabolic conditions of animals, including egg-laying hens.

## Materials and Methods

This study was performed in accordance with the Chinese guidelines for animal welfare and experimental protocols. It was approved by the China Agricultural University Animal Experiment Ethics Committee (CAU20160916-2).

### Birds and Husbandry

A total of 688 Rhode Island White hens were raised by Beijing Huadu Yukou Poultry Industry Co. Ltd. (Yukou, China). During the rearing period, each bird was fitted with a leg ring marked with a unique identification number and raised in individual cages (cage size: 45 cm × 45 cm × 45 cm). They were acclimated to the environment and diet. During the laying period, egg production was recorded daily for each hen. These hens were fed a basal diet that was formulated to meet the National Research Council requirements (27); it was offered in mash form *ad libitum*. The composition and nutrient levels of the basal diet are shown in Supplementary Table 1. Water was supplied by nipple drinkers. The room temperature was maintained between 22 and 26°C, and light exposure was controlled with a light:dark cycle of 16 h:8 h. Illumination was provided by incandescent lamps with an intensity of 10 lx (at the head level of the birds). All procedures as well as the care, housing, and handling of the animals were conducted according to accepted commercial management practices. All the birds remained healthy during the rearing period. No birds were culled, and none received any medical intervention.

At 80 weeks of age, the blood samples of these layers were collected for genotype analysis (A/T polymorphism at position 1034 of chicken *FMO3* exon 7, chromosome 8; accession number: AJ431390). A polymerase chain reaction restriction fragment length polymorphism assay as described by Zhang (28) was used to determine the individual *FMO3* genotypes (AA, AT, and TT) at this position. Subsequently, a total of 525 AA, 154 AT, and nine TT hens were obtained from the whole flock.

### Sampling

At 90 weeks of age, after a 10-h fast, 27 *FMO3* genotyped individuals (nine AA hens, nine AT hens, and nine TT hens) with normal laying performance were selected. Their blood samples were collected during the morning. Blood samples were stored in vacuum blood collection tubes containing ethylenediaminetetraacetic acid. The plasma was separated by centrifugation at 3,000 × g for 15 min and stored at −20°C until analysis. Then, the 27 birds were humanely euthanized. The liver, abdominal fat, and wet weight of ovaries were measured to calculate the organ indices, and the subcutaneous fat thickness of the back was measured. Subsequently, one part of the liver tissue samples was collected, frozen in liquid nitrogen, and stored in a freezer (−80°C); it was used to obtain measurements of antioxidative indices and triglyceride (TG) levels. The other parts of the liver samples were fixed for histopathological observation. The aorta macrovessels were also isolated and fixed in formalin for 48 to 72 h before being processed to analyze AS lesions.

Additionally, one healthy AA pullet (18 weeks old) was euthanized, and its whole aorta and liver tissues were isolated in the same manner. These samples were used as a negative control for the AS lesion analysis and liver histopathological observations because our pre-experiment indicated that lipid droplets were rarely observed in the aortic wall and liver of pullets at 18 weeks.

### Plasma Index Measurement

Plasma total cholesterol (TC), low-density lipoprotein cholesterol (LDL-C), TG, glucose (Glu), alanine aminotransferase (ALT), aspartate aminotransferase (AST), and creatinine (CRE) levels were determined using commercial kits (Shanghai Kehua Bioengineering Co., Ltd., Shanghai, China). The KHB ZY-1280 automatic biochemical analyzer (Shanghai Kehua Bio-engineering Corporation, Shanghai, China) was used. Plasma very low-density lipoprotein (VLDL), free fatty acid (FFA), insulin (INS), interleukin (IL)-1β, IL-6, and IL-8 levels were measured using chicken VLDL, FFAs, INS, IL-1β, IL-6, and IL-8 enzyme-linked immunosorbent assay kits (VLDL ELISA kit JLC10779, FFA ELISA kit JLC10804, INS ELISA kit JLC10935, IL-1β ELISA kit JLC10840, IL-6 ELISA kit JLC10846, and IL-8 ELISA kit JLC10848; Shanghai Jingkang Bioengineering Co., Ltd., Shanghai, China) according to the manufacturer's instructions.

### Lipid Deposition Characteristics

#### AS Lesions Assessment

AS lesions were quantified by performing an en face analysis of the aorta (including the aortic arch, thoracic region, and abdominal region) and cross-sectional analysis of the aortic arch, as previously described by Chen et al. (29) and Collins et al. (30), with minor modifications. During the en face analysis, the aorta was longitudinally opened and stained with Oil Red O (Wuhan Service Biotechnology Co., Ltd., Wuhan, China) to detect lipids and determine the lesion area. AS lesions of the aorta are expressed as percentages of the total surface area. During the cross-sectional analysis, a small segment of the aortic arch (in the same area) was embedded in OCT compound (Sigma-Aldrich, St. Louis, MO, USA) and frozen at −20°C. Sections (thickness, 8 μm) were collected. Lesions from 10 alternating sections were stained with Oil Red O and hematoxylin. For each section of the aortic arch, 10 randomly selected areas were assessed using light microscopy at ×20 magnification.

#### Liver Pathological Observation and Scoring System

Liver sections were examined for steatosis using Oil Red O staining, as previously described by Gao et al. (31), with modifications. To perform cryosection cutting, fixed samples were embedded in frozen OCT (Sigma-Aldrich, St. Louis, MO, USA) and sectioned at 10 μm; all procedures were performed under frozen conditions. Then, samples were stained with Oil Red O (Wuhan Service Biotechnology Co., Ltd., Wuhan, China), differentiated with isopropanol, washed with distilled water, and stained with hematoxylin. Fat vacuoles in hepatocytes were stained red by Oil Red O, and cell nuclei were stained black and blue with hematoxylin. For each section of the liver, 10 randomly located areas were assessed using light microscopy at ×80 magnification.

Pathological observations of the liver were divided into five grades as previously described by Lv et al. (32), with a score of 0 indicating the normal state, a score of 0.5 indicating lipid deposition was between 0 and 1 points, a score of 1 indicating that fat vacuoles in the liver cells were small and scattered, a score of 2 indicating that fat vacuoles in the liver cells were large and wide, and a score of 3 indicating that fat vacuoles were fused into large vacuoles and the nucleus was squeezed into the cell membrane, similar to the adipocytes. The specific clinical categorization scheme used for assessing FLS is shown in Supplementary Table 2.

Images of the aortic en face, aortic arch cross-sections, and liver sections were obtained using a Canon EOS 7D digital camera (Canon, Tokyo, Japan). The aorta, aortic arch, and liver lesions were quantified using computer-assisted image analysis (ImageJ version 1.8.0; NIH Image, National Institutes of Health, Bethesda, MD, USA) according to the procedures described by Schneider et al. (33).

### Hepatic TG and Antioxidant Indices

Hepatic TG levels were determined using kits (TG detection kit A110-2-1; Nanjing Jiancheng Bioengineering Institute, Nanjing, China). Hepatic glutathione peroxidase (GSH-Px), total superoxide dismutase (T-SOD), and catalase (CAT) activity were also determined using kits (GSH-Px detection kit A005, SOD detection kit A001-3, and CAT detection kit A0071-1; Nanjing Jiancheng Bioengineering Institute, Nanjing, China). Hepatic malondialdehyde (MDA) levels were determined using a thiobarbituric acid assay (MDA detection kit A003-1; Jiancheng Bioengineering Institute, Nanjing, China). All indices were measured according to the manufacturer's instructions. The protein concentrations of the samples were measured using the Bradford method (34).

### Organ Indices

Organ indices were calculated using the following formulas: liver index (LI) = liver weight (g)/body weight (g) × 100%; abdominal fat rate (AFR) = abdominal fat (g)/body weight (g) × 100%; and ovarian index (OI) = organ weight (g)/body weight (g) × 100%.

### Statistical Analysis

Statistical analyses were conducted using R software (version 4.0.3; The R Foundation for Statistical Computing, Vienna, Austria), and the figures were plotted using GraphPad Prism (version 7.04; GraphPad Software, San Diego, CA, USA). Because of the small sample size, non-parametric procedures were performed. An independent sample Kruskal-Wallis test was used to analyze the plasma metabolic indices, phenotypic traits (fat deposition characteristics in the aorta, liver, subcutis, abdomen, organ indices, and production performance), and hepatic indices. Correlations between plasma metabolic indices and phenotypic traits as well as correlations between hepatic indices and phenotypic traits were analyzed using Pearson's correlation coefficient and “ggcorrplot” package in R.

## Results

### Histopathology Assessment of Aorta

**Figure 1:**
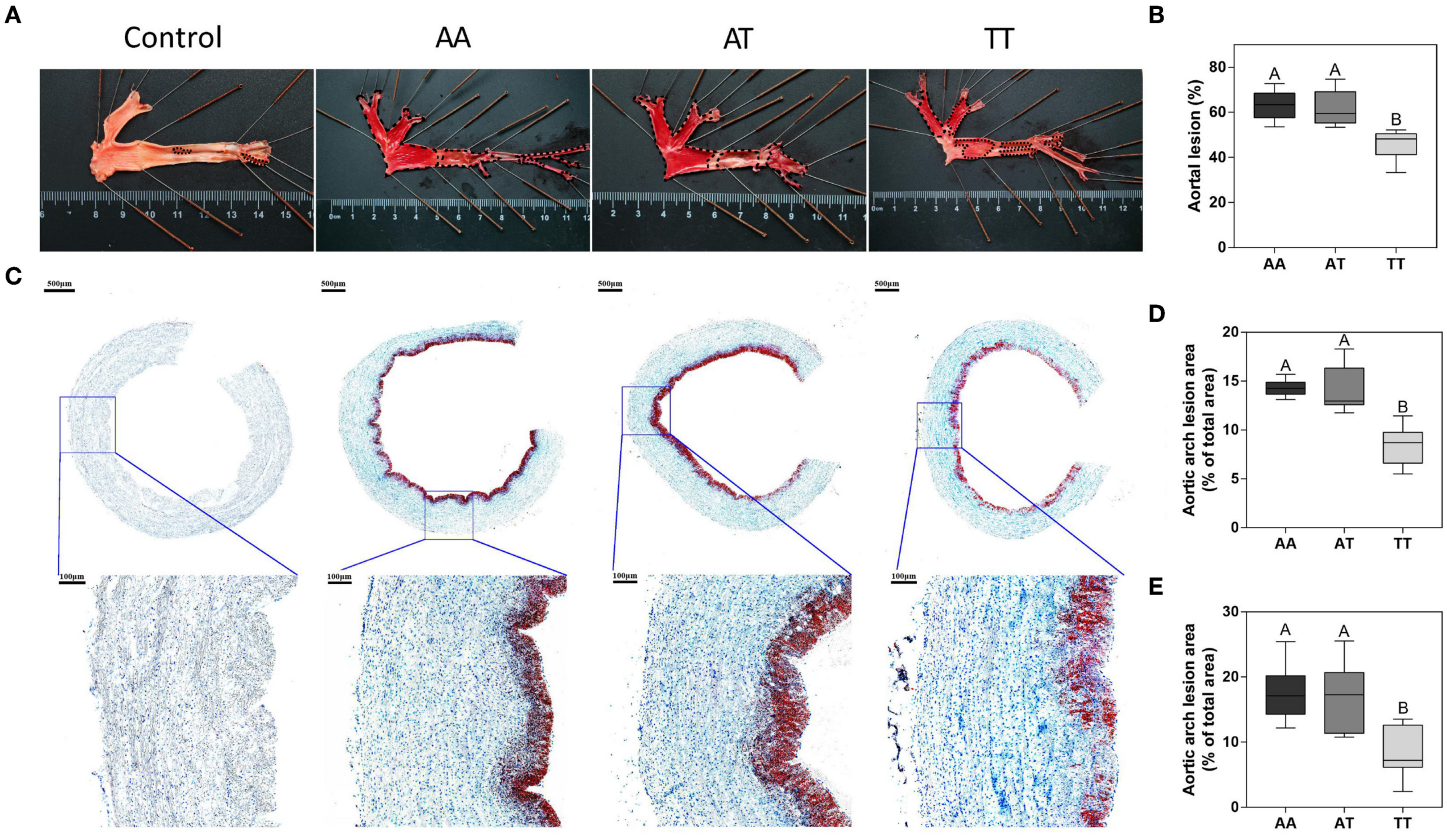
Pathological observations of lipid deposition in the aortic wall in different genotyped layers. **(A)** Oil Red O staining of the whole aorta with aortic lesions (black dashed lines). **(B)** Quantification of staining results (% of the total area) of the aorta. **(C)** Oil Red O and hematoxylin staining of aortic arch cross-sections with local tissue magnification (blue squares). Quantification of staining results (% of the total area) of the aortic arch cross-section **(D)** and its local tissue magnification (blue squares) **(E)**. **(B,D,E)** Box plot lower extreme is first quartile; box plot upper extreme is third quartile. AA, AA genotype hens; AT, AT genotype hens; TT, TT genotype hens. *n* = 9 hens with each genotype. ^A,B^Means within a histogram with no common superscripts differ significantly (*P* < 0.01).

The histopathological assessment of AS for the AA, AT, and TT genotypes is shown in Figure 1. As expected, in TT hens, T329S significantly decreased (*P* < 0.01) the aortic lesion (AL) areas by 27% compared to AA hens (Figure 1A) and by 26% compared to AT hens (Figure 1B). Furthermore, in TT hens, it decreased (*P* < 0.01) the aortic arch lesion (AAL) proportion by 41% compared to AA hens (Figure 1C) and by 40% compared to AT hens (Figure 1D). Concordant with this, TT hens had the least (*P* < 0.01) lipid droplet accumulation in the intima and media of the vessel wall among the three genotyped layers (Figure 1E).

### Plasma Metabolic Characteristics

The plasma metabolic characteristics of the layers with AA, AT, and TT genotypes are shown in Table 1. As an effect of the T329S mutation, the plasma TC and LDL-C levels showed a decreasing trend, and the plasma VLDL levels were decreased (*P* < 0.05); however, the plasma TG levels were not changed in TT hens when compared to those of AA and AT hens. Additionally, T329S decreased the plasma FFA and INS levels (*P* < 0.05); however, T329S increased the plasma Glu levels (*P* < 0.05) of AT and TT hens compared to that of AA hens. Furthermore, it decreased the plasma IL-1β, IL-6, and IL-8 levels of AT and TT hens compared to those of AA hens, and it especially decreased plasma IL-1β and IL-8 levels (*P* < 0.05) of TT hens compared to those of AA hens. Additionally, T329S significantly (*P* < 0.05) decreased the plasma ALT, AST, and CRE levels of TT hens compared to those of AA and AT hens.

**Table 1:** Plasma metabolic characteristics of layers with different *FMO3* genotypes.

**Genotype**	**AA**	**AT**	**TT**	**Total**
				***P*-value**
**Metabolic indices** ^**1**^				
TC (mmol/L)	3.81 ± 2.03	3.96 ± 1.72	3.60 ± 1.32	*P* > 0.05
LDL-C (mmol/L)	1.38 ± 0.86	1.45 ± 0.68	1.30 ± 0.75	*P* > 0.05
VLDL (mmol/L)	8.35 ± 4.71^a^	7.07 ± 5.60^a^	5.38 ± 4.60^b^	*P* < 0.05
TG (mmol/L)	10.61 ± 1.01	10.58 ± 1.00	10.60 ± 1.37	*P* > 0.05
FFAs (μmol/L)	629.79 ± 199.63^a^	463.75 ± 84.83^b^	483.93 ± 36.63^b^	*P* < 0.05
INS (mU/L)	67.35 ± 31.28^a^	56.24 ± 20.10^b^	55.73 ±19.46^b^	*P* < 0.05
Glu (mmol/L)	13.29 ± 0.82^b^	14.74 ± 1.93^a^	14.66 ± 1.36^a^	*P* < 0.05
**Inflammatory cytokines** ^**2**^				
IL-1β (pg/mL)	103.89 ± 20.82^a^	97.99 ± 6.61^ab^	96.68 ±13.89^b^	*P* < 0.05
IL-6 (pg/mL)	21.66 ±13.37	18.75 ± 9.84	18.84 ±11.47	*P* > 0.05
IL-8 (pg/mL)	88.13 ± 46.37^a^	74.51 ±11.31^ab^	66.53 ± 8.57^b^	*P* < 0.05
**Liver and kidney injury indicators** ^**3**^				
ALT (U/L)	32.59 ± 5.83^a^	34.71 ± 7.68^a^	29.75 ± 6.10^b^	*P* < 0.05
AST (U/L)	206.75 ± 47.48^a^	202.64 ± 95.23^a^	173.47 ± 17.51^b^	*P* < 0.05
CRE (mmol/L)	45.74 ± 6.30^a^	45.59 ± 3.95^a^	41.82 ± 5.01^b^	*P* < 0.05

1 *TC, total cholesterol; LDL-C, low-density lipoprotein cholesterol; VLDL, very low-density lipoprotein; TG, triglyceride; FFAs, free fatty acids; INS, insulin; Glu, glucose*;

2 *IL-1β, interleukin-1β; IL-6, interleukin-6; IL-8, interleukin-8*;

3 *ALT, alanine aminotransferase; AST, aspartate aminotransferase; CRE, creatinine; AA, AA genotype hens; AT, AT genotype hens; TT, TT genotype hens. Values are expressed as means ± standard deviation (SD); n = 9 hens with each genotype*.

a,b *Means within a row with no common superscript differ significantly (P < 0.05)*.

### Histopathology Assessment of the Liver

**Figure 2:**
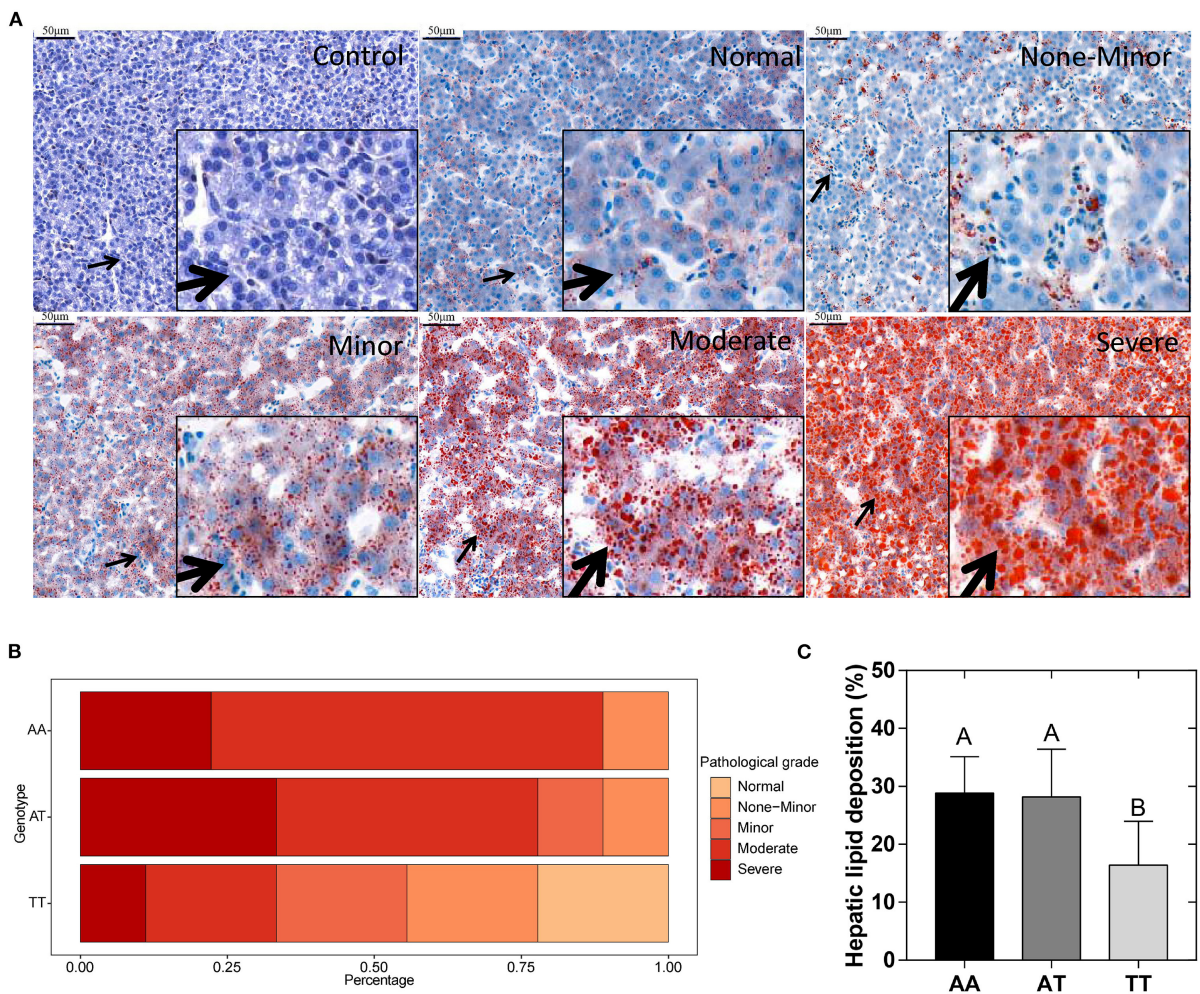
**(A)** Histopathologic observations of chicken livers were divided into five grades representing the different degrees of fatty liver syndrome (arrows point to local tissue magnification). **(B)** Incidence rates of different grades of fatty liver syndrome in different genotyped layers. **(C)** Quantification of Oil red O staining results (% of the total area) of the liver. AA, AA genotype hens; AT, AT genotype hens; TT, TT genotype hens. Values are expressed as means ± standard deviation (SD); *n* = 9 hens with each genotype. ^A,B^Means within a histogram with no common superscripts differ significantly (*P* < 0.01).

**Figure 3:**
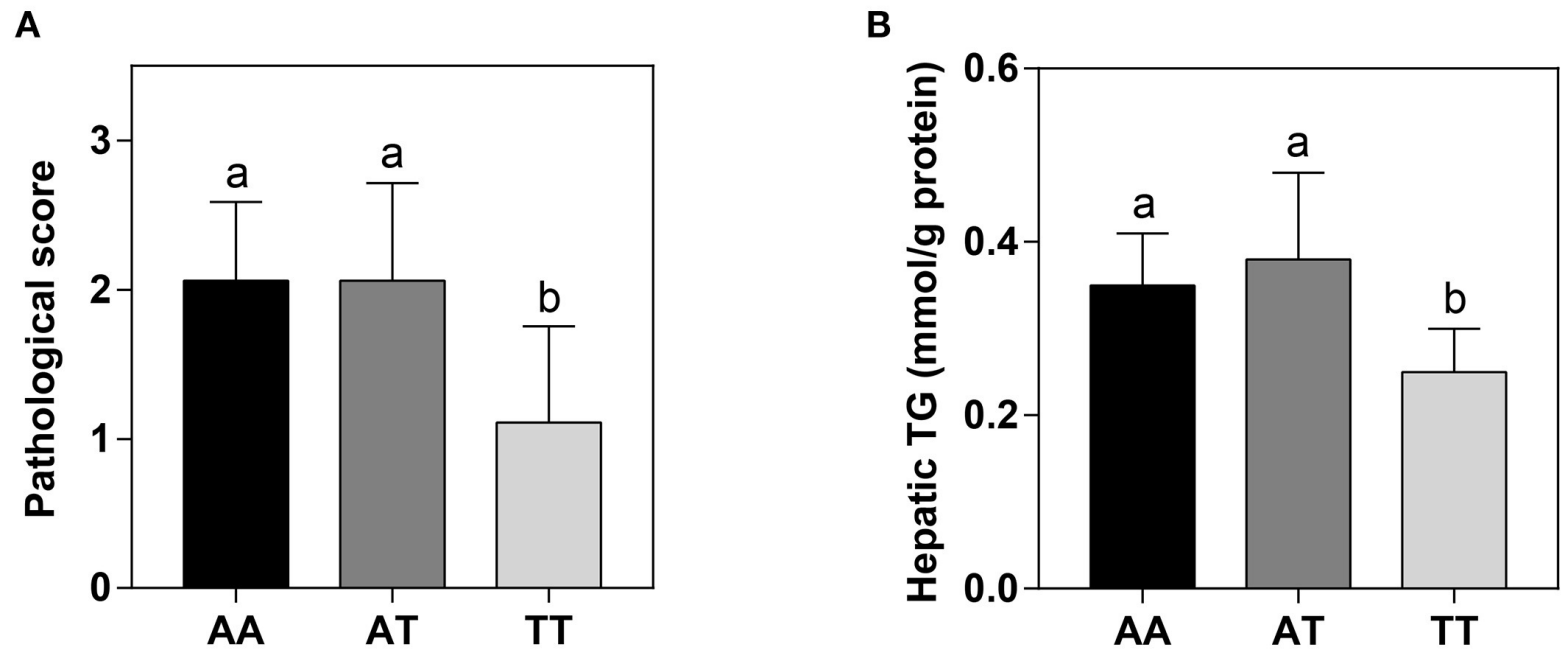
Hepatic pathological scores **(A)** and TG levels **(B)** in different genotyped layers. TG, triglyceride; AA, AA genotype hens; AT, AT genotype hens; TT, TT genotype hens. Values are expressed as means ± standard deviation (SD); *n* = 9 hens with each genotype. ^a,b^Means within a histogram with no common superscripts differ significantly (*P* < 0.05).

The hepatic histological observations, pathological grades, and lipid deposition of layers with the AA, AT, and TT genotypes are shown in Figure 2. The five grades of FLS severity determined with Oil Red O staining are shown in Figure 2A. We defined the moderate (score 2) and severe (score 3) grades as FLS. Interestingly, the T329S mutation reduced the incidence of FLS in TT hens, which was less than half that of AA and AT hens, which showed that TT hens were more inclined to have normal and minor grades (Figure 2B). Concordant with this, TT hens had the least (*P* < 0.01) hepatic lipid deposition (HLD) (Figure 2C) and the lowest (*P* < 0.05) hepatic pathological scores and hepatic TG levels among the three genotyped layers (Figures 3A,B).

### Hepatic Antioxidant Indices

**Figure 4:**
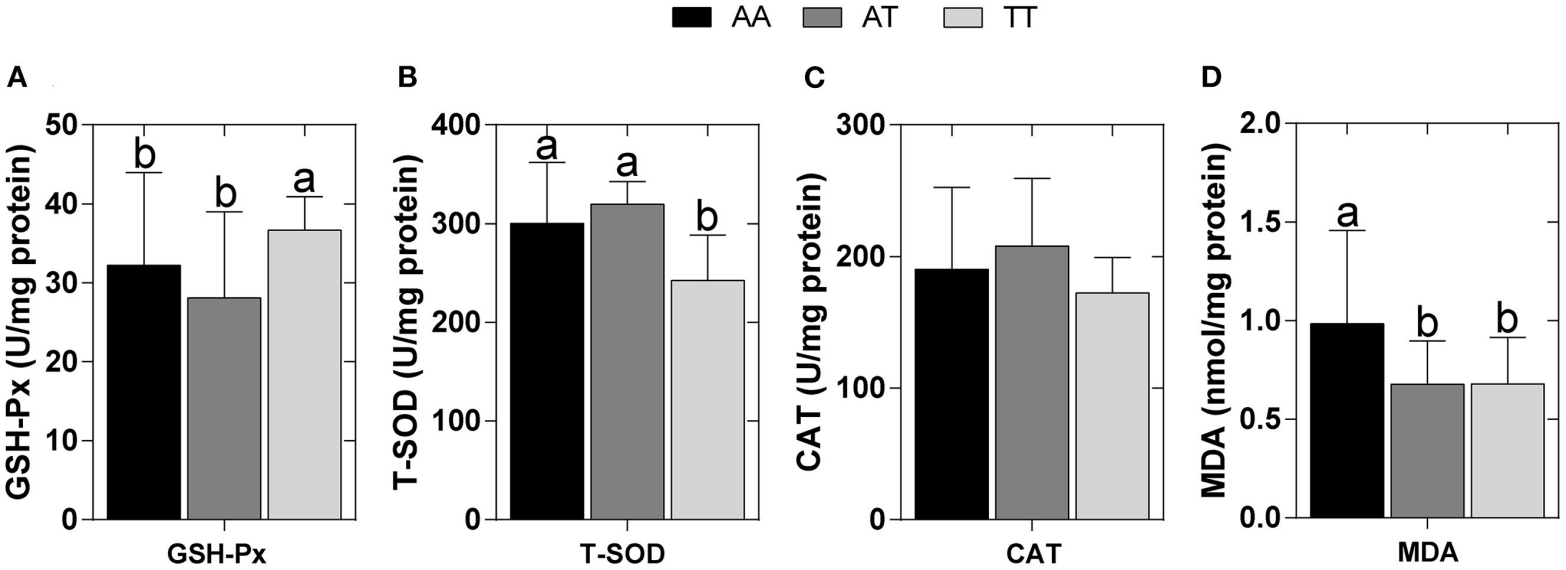
Hepatic antioxidant indices in different genotyped layers. **(A)** Glutathione peroxidase (GSH-Px) activity; **(B)** Total superoxide dismutase (T-SOD) activity; **(C)** Catalase (CAT) activity; **(D)** Hepatic malondialdehyde (MDA) levels. AA, AA genotype hens; AT, AT genotype hens; TT, TT genotype hens. Values are expressed as means ± SD, *n* = 9 hens each genotype. ^*a, b*^Means within a histogram with no common superscripts differ significantly (*P* < 0.05).

The hepatic antioxidant indices of the layers with AA, AT, and TT genotypes are shown in Figure 4. As a result of the T329S mutation, in TT hens, the hepatic GSH-Px activity was increased by approximately 14% compared to that of AA hens and by approximately 30% compared to that of AT hens (*P* < 0.05) (Figure 4A). Furthermore, the hepatic T-SOD activity in TT hens was decreased by approximately 19% compared to that of AA hens and by approximately 24% compared to that of AT hens (*P* < 0.05) (Figure 4B); however, T329S did not alter the activity of hepatic CAT in layers (Figure 4C). Moreover, hepatic MDA levels were decreased by approximately 45% (*P* < 0.05) in AT and TT hens compared to that in AA hens (Figure 4D).

### Organ Indices and Egg Production

**Figure 5:**
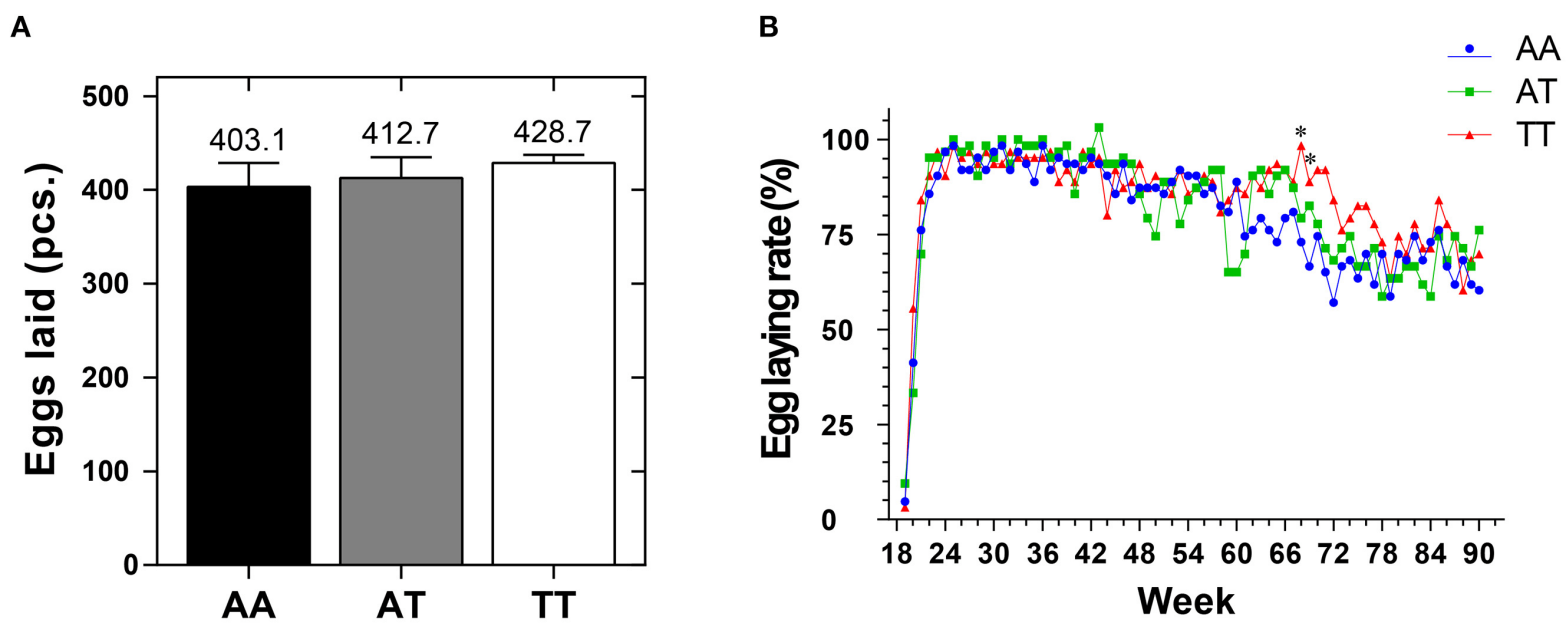
The average number of eggs **(A)** and the egg-laying rate **(B)** of sample layers with different *FMO3* genotypes from 19 to 90 weeks. *FMO3*, flavin-containing monooxygenase 3. AA, AA genotype hens; AT, AT genotype hens; TT, TT genotype hens. Values are expressed as means ± standard deviation (SD); *n* = 9 hens with each genotype. Mean values with (*) differ significantly between the AA and TT hens (*P* < 0.05).

The organ indices of the different genotyped layers at 90 weeks are shown in Table 2. The T329S mutation reduced (*P* < 0.05) the LI more in TT hens than in AA and AT hens. Additionally, T329S reduced (*P* < 0.05) the AFR and subcutaneous fat thickness (SFT) more in AT and TT hens than in AA hens; however, it did not alter (*P* > 0.05) the OI of layers. Additionally, it increased the average number of eggs laid by layers (EN90) from 19 to 90 weeks and the egg-laying rate after 68 weeks in TT hens more than it did in AA and AT hens (Figures 5A,B).

**Table 2:** Effects of the T329S mutation on organ indices in layers.

**Item^**1**^**	**AA**	**AT**	**TT**	**Total**
				***P*-value**
Liver index (%)	1.86 ± 0.25^a^	2.02 ± 0.49^a^	1.62 ± 0.24^b^	*P* < 0.05
Abdominal fat rate (%)	6.83 ± 1.73^a^	5.10 ± 1.77^b^	5.42 ± 0.96^b^	*P* < 0.05
Subcutaneous fat	10.99 ± 2.46^a^	9.46 ± 2.43^b^	9.50 ± 2.42^b^	*P* < 0.05
thicknesses (mm)				
Ovarian index (%)	1.88 ± 0.74	2.31 ± 0.75	1.94 ± 0.32	*P* > 0.05

1 *Liver index (%) = liver weight (g)/body weight (g) × 100%; Abdominal fat rate (%) = abdominal fat (g)/body weight (g) × 100%; and ovarian index (%) = organ weight (g)/body weight (g) × 100%. AA, AA genotype hens; AT, AT genotype hens; TT, TT genotype hens. Values are expressed as means ± standard deviation (SD); n = 9 hens with each genotype*.

a,b *Means within a row with no common superscript differ significantly (P < 0.05)*.

### Correlations Between Metabolic Indices and Phenotypic Traits

**Figure 6:**
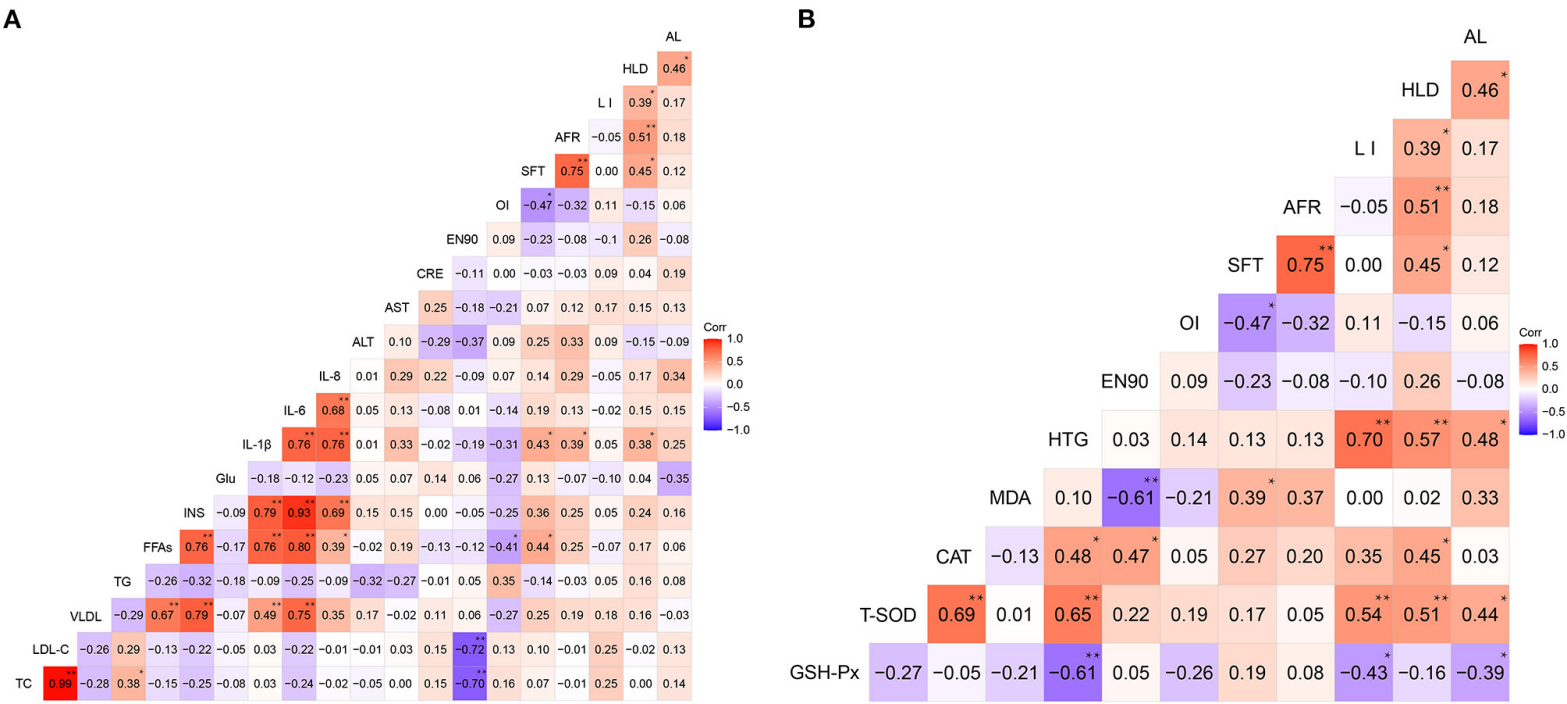
Heat map based on Pearson's correlations for the relationships among metabolic indices and phenotypic traits (fat deposition characteristics in the aorta, liver, subcutis, and abdomen, organ indices, and production performance) of the 27 selected hens at 90 weeks. **(A)** Correlations between plasma metabolic indices and phenotypic traits. **(B)** Correlations between hepatic metabolic indices and phenotypic traits. **(A)** Correlations between plasma metabolic indices and phenotypic traits. **(B)** Correlations between hepatic metabolic indices and phenotypic traits. The color scale represents Pearson's correlation coefficients, with red and bluish violet representing positive and negative correlations, respectively. Ranging from 1.0 (maximum positive correlation) to −1.0 (maximum anti-correlation), with 0 indicating no correlation. TC, total cholesterol; LDL-C, low-density lipoprotein cholesterol; VLDL, very low-density lipoprotein; TG, triglyceride; FFAs, free fatty acids; INS, insulin; Glu, glucose; IL-1β, interleukin-1β; IL-6, interleukin-6; IL-8, interleukin-8; ALT, alanine aminotransferase; AST, aspartate aminotransferase; CRE, creatinine; EN90, average number of eggs laid by layers from 19 to 90 weeks; OI, ovarian index; SFT, subcutaneous fat thickness; AFR, abdominal fat rate; LI, liver index; HLD, hepatic lipid deposition; AL, aortic lesion; GSH-Px, glutathione peroxidase; T-SOD, total superoxide dismutase; CAT, catalase; MDA, malondialdehyde; HTG, hepatic triglyceride. *P < 0.05, **P < 0.01.

The correlation matrices of the relationships between plasma metabolic indices and phenotypic traits (fat deposition characteristics in the aorta, liver, subcutis, abdomen, organ indices, and production performance) as well as the relationships between hepatic indices and phenotypic traits are shown in Figure 6. Of the 27 hens, positive correlations among AL, HLD, AFR, and SFT were detected. Furthermore, these fat deposition traits were positively correlated with plasma IL-1β, IL-6, and IL-8 levels. In particular, the HLD (*r* = 0.38), AFR (*r* = 0.39), and SFT (*r* = 0.43) were significantly (*P* < 0.05) correlated with plasma IL-1β levels (Figure 6A). Furthermore, the AL, HLD, and LI were negatively correlated with hepatic GSH-Px activities; however, they were positively (P < 0.05) correlated with hepatic T-SOD activity (Figure 6B). These results illustrated the association between lipid deposition traits and metabolic indices of the plasma and liver of the layers.

Additionally, a significant (*P* < 0.05) negative correlation between the EN90 and plasma cholesterol, including TC (*r* = −0.70) and LDL-C (*r* = −0.72), was detected in the layers (Figure 6A). A negative correlation between the EN90 and hepatic MDA levels (*r* = −0.61; *P* < 0.05) (Figure 6B) and negative correlations (*P* < 0.05) between the OI and FFAs (*r* = −0.41) as well as SFT (*r* = −0.47) were detected (Figure 6A). These results confirmed that production performance is associated with changes in plasma and hepatic metabolic indices. They also imply that lipid deposition or adiposity-related diseases could further affect the production performance of layers.

## Discussion

During this study, we confirmed that the spontaneous genetic mutation T329S in *FMO3* could reduce AS lesions by causing changes in metabolic indices in TT hens. This result clarifies the relationship among *FMO3*, AS, and metabolic characteristics in chicken models. Furthermore, we were surprised to find that the T329S mutation reduced the incidence of FLS and fat deposition in the subcutis and abdomen of layers. These results were consistent with our hypothesis and implied that T329S may create a better health status for older adults. Moreover, a correlation analysis of metabolic indices and phenotypic traits further demonstrated the effects of T329S on the metabolic and phenotypic characteristics of laying hens. Our current study provides evidence to support the association between *FMO3* and lipid metabolic diseases. It could also provide a new strategy for using T329S to improve the health status and production performance of layers during the late laying period.

### Effects of *FMO3* Genotypes on AS

Numerous studies of mammals have revealed a significant association between the TMA/FMO3/TMAO pathway and AS. Furthermore, experiments involving TMAO supplementation for mice have provided evidence that TMAO is a risk factor for AS (7, 35, 36). Subsequently, several studies involving humans and mice have confirmed that a reduction in endogenous TMAO concentrations reduces AS and vascular inflammation (9, 29). During the present study, our results showed that the T329S mutation in *FMO3* decreased the AL and AAL in TT hens compared to those in AA and AT hens at 90 weeks. This result is consistent with our previous observations, which showed that T329S reduced AS lesions in a strain of chickens laying brown eggs at 62 weeks (19). This result can be attributed to T329S diminishing the activity of the TMAO-generating enzyme FMO3 and therefore decreased the plasma circulating TMAO concentrations in TT hens. Because Guo (17) has demonstrated that TT hens have the lowest (*P* < 0.05) FMO3 activity among the three genotypes and Wang et al. (16) have also shown that the TT hens have a lower plasma circulating TMAO concentrations than those of AA hens (~150 μg/mL vs. ~190 μg/mL, *P* < 0.05). TMAO has been shown to suppress reverse cholesterol transport and affect lipid absorption and cholesterol homeostasis by inhibiting bile acid synthesis, and to subsequently induce cholesterol accumulation in macrophages, finally resulting in the development of AS (7–9, 37). In contrast, a decrease in the plasma TMAO concentration associated with an increase in the bile acid pool has been suggested as a possible mechanism of the T329S mutation involved in the reduction of AS lesions in chickens (19). Therefore, these results confirmed that the spontaneous mutation in *FMO3* could alleviate AS lesions in layers. Previous studies have noted that the occurrence and development of AS are spontaneous processes in older chickens, and that their present lipoprotein levels are similar to those of humans. Therefore, chickens have been considered a good animal model for the study of AS (38). Subsequently, chickens with the T329S mutation could provide a reliable and spontaneous mutation for further studies.

### Effects of *FMO3* Genotypes on Plasma Indices

Several potential mechanisms by which the FMO3 pathway associates with AS have been identified, including alterations in cholesterol and lipid metabolism, Glu metabolism, diabetes-related traits, and vascular inflammation (7, 9, 11, 39). The indices in plasma could directly reflect changes in these metabolic characteristics (13). During this study, our results showed that the T329S mutation caused decreases in plasma TC, LDL-C, and VLDL levels in TT hens. These results implied that the T329S mutation could alter cholesterol and lipid metabolism of laying hens. The decreasing trends of plasma TC and LDL-C levels of TT hens are consistent with our previous findings, which showed that T329S moderated the serum lipid parameters of TT hens compared to those of AA and AT hens from 49 to 62 weeks (19). It is known that higher plasma TC and LDL-C levels could increase the risk of AS; however, modulating these cholesterol levels can reduce the incidence of diseases in older laying hens (32, 40). Accordingly, the decreased trends of TC and LDL-C levels of TT hens explained the decrease in AS lesions. However, the observed trend of plasma VLDL levels of TT hens is inconsistent with that of our previous study, which showed that TT hens have higher VLDL levels at 62 weeks (19). This might be related to hepatic TG secretion because VLDL is the major transport vehicle for TG from the liver to extrahepatic tissues, or it might be attributable to the dynamic change of VLDL subclasses in layers during the normal aging process (25, 41). These hypotheses remain to further study.

Our results suggested an association between T329S and Glu metabolism and lipogenesis in layers because a significant (*P* < 0.05) decrease in the plasma FFA and INS levels and an increase in the plasma Glu levels were detected in TT hens (Table 1). It is known that FFAs can be derived from lipolysis in adipocytes and *de novo* lipogenesis in hepatocytes, and that INS can stimulate the uptake of Glu, transport fatty acids into adipocytes, and promote lipogenesis (42, 43). Therefore, the decrease in plasma FFA and INS levels and the increase in Glu levels in TT hens could imply that T329S reduced the consumption of Glu and lipogenesis in the layers. In parallel, similar trends of these plasma indices were also observed in AT hens (Table 1), thus supporting this claim. Additionally, our study detected a significant (*P* < 0.05) decrease in indicators of inflammation (e.g., IL-1β and IL-8) and liver and kidney injury (e.g., ALT, AST, and CRE) in TT hens compared to those in AA hens (Table 1). These proinflammatory factors released during an immune response could result in inflammation and subsequent metabolism anomalies that often lead to tissue injury (44). In contrast, the decreasing trends of inflammatory indicators are consistent with the trends of AS in TT hens (Figure 1), suggesting that T329S decreases the inflammatory response caused by lipid deposition. The decrease in indicators of liver and kidney injury in TT hens may further imply an association between T329S and other lipid metabolic factors related to liver and kidney damage, such as FLS and adiposity (32, 45). Collectively, these results confirmed the effects of the T329S mutation on AS lesions and plasma metabolic indices of 90-week-old layers.

### Effects of FMO3 Genotypes on Incidence of FLS

In chickens, lipid metabolic conditions associated with AS lesions, including FLS and adiposity, mainly occur during the late laying period. The occurrence and development of FLS and adiposity are primarily caused by continuous egg production, high consumption of dietary carbohydrates, and the subsequent imbalance between deposition and the removal of lipids in older layers (20, 23, 24). Among these conditions, FLS has the most serious impact on the chicken industry because the liver is the main organ involved in the formation of yolk precursors in layers (25, 26). More seriously, FLS can induce sudden death (32). For example, during the laying cycle of commercial layers, FLS caused up to 5% mortality and 74% of the total mortality of caged laying hens in Queensland (46). During our study, we were surprised to find that the TT hens had a lower incidence of FLS, lower (*P* < 0.05) hepatic pathological scores and TG deposition, and lower (*P* < 0.05) LI compared to those of AA and AT hens (Figures 2A–C, 3A,B, Table 2). These results indicated that the T329S mutation was associated with a decrease in hepatic lipid deposition and the incidence of FLS in layers, which implied that T329S could protect the liver from injury. To our knowledge, this finding and the molecular mechanisms involved have not been previously reported for layers. We speculated that this finding could be attributed to two possible mechanisms. First, T329S induced a decrease in circulating TMAO concentrations, which subsequently improved reverse cholesterol transport in TT hens (mentioned previously) because TMAO has been associated with adverse effects on FLS and liver inflammation and damage in humans (47). Second, T329S is associated with decreases in plasma FFA and INS levels because a high FFA level can deteriorate INS sensitivity, thus creating a vicious cycle between hyperinsulinemia and HTG levels (48). Hence, the observed decrease in the incidence of FLS in TT hens could be attributed to the combined action of the two possible mechanisms. However, the precise molecular mechanism requires further study.

Furthermore, the “two-hit hypothesis” of FLS states that fat deposition caused by abnormal fatty acid metabolism in the liver represents the “first hit,” and that increased levels of INS, inflammatory cytokines, and oxidative stress induced by fat deposition represent the “second hit” (49–51). The levels of antioxidant enzymes, such as GSH-Px, T-SOD, and CAT, and the oxidative biomarker MDA could further evaluate oxidative damage in the liver (52). During our study, TT hens had higher hepatic GSH-Px activity but lower hepatic T-SOD activity and a lower level of hepatic MDA than those in AA hens (*P* < 0.05) (Figure 4), suggesting that T329S is associated with hepatic oxidative damage of layers. GSH-Px and T-SOD are normally used to scavenge reactive oxygen species, a class of substances that can disrupt the formation of biofilm, and therefore protect cells from oxidative damage (52, 53). MDA is one of the final products of lipid oxidation and is strongly toxic to cells (52). Therefore, the increased activity of hepatic GSH-Px and the decrease in hepatic MDA levels could reduce oxidative injury in TT hens. The observed activity of hepatic T-SOD was increased in TT hens; however, it was decreased in AA hens. This could be attributed to a compensatory mechanism of the antioxidant defense system against increased oxidative stress because hepatic T-SOD was positively correlated with AL, HLD, LI, and HTG in 27 hens (Figure 6). These results further suggest that the T329S mutation in *FMO3* has a protective effect on the liver and confirm the role of T329S in alleviating the development of FLS in older layers.

In addition, we also found that the hepatic lipid deposition levels exist deviation to some extent within the group (Figures 2B,C). This phenomenon might be attributed to the effects of genetic factors and individual differences, because hepatic lipid deposition is a complex trait that is regulated by multiple co-varied factors (54). For example, the alterations in the AMP-activated protein kinase (AMPK). AMPK dephosphorylation causes fatty acid synthesis (lipogenesis) through the dephosphorylation of acetyl-CoA carboxylase (ACC) and up-regulation of sterol regulatory element binding protein. Then, ACC transforms acetyl-CoA to malonyl-CoA. Elevated malonyl-CoA inhibits CPT1 activity (related with fatty acid oxidation), resulting in the development of FSH (55). The exact reason for this deviation within the group should be further explored. Collectively, our current results suggested, in part, that the T329S mutation could decrease the incidence of FLS in TT hens.

### Effects of FMO3 Genotypes on Adiposity and Performance

Our current results also showed that T329S was associated with a reduction in adiposity in older layers because it decreased the AFR and SFT ratios in AT (*FMO3*, c. 984 A > T) and TT hens at 90 weeks (Table 2). This finding, combined with our results regarding AS and FLS, demonstrated that the T329S mutation could improve lipid metabolic conditions of layers during the late laying period. This may imply that T329S could improve the laying performance because lipid metabolic diseases are challenging in older layers (23). Furthermore, our results showed that the EN90 and egg-laying rate after 68 weeks were increased in TT hens (Figures 5A,B). This result can be attributed to the fact that T329S improved the plasma indices and decreased fat deposition and lipid peroxidation in TT hens because prominent (*P* < 0.05) negative correlations among EN90 and TC, LDL-C, and MDA and negative correlations among OI and FFAs and SFT were detected during our study (Figure 6). In brief, these results suggest that T329S could decrease the incidence of FLS and body fat deposition and are associated with improvements in the laying performance of older layers.

These results have profound implications for the poultry industry. They imply that the polymorphisms diminishing the activity of FMO3 were associated with the improvement of lipid metabolic conditions in the layers. These conditions threaten the health status and laying performance of older layers and can lead to a sudden decrease in egg production during the late laying period (23). The decreased performance in chickens would negatively impact the poultry industry (23, 56). Accordingly, our results suggest that the T329S mutation or similar methods of inhibiting FMO3 enzyme activity could be used to alleviate these conditions and subsequently improve the laying performance of older layers, such as TT hens with T329S or AA and AT hens with inhibited FMO3 enzyme activity used as breeder hens to improve the egg-producing efficiency of breeder flocks. These methods may increase the TMA levels in eggs when the layers are fed a precursor diet with a high level of TMA (5). The changes in TMA levels of eggs do not impair their hatching rate (57).

## Conclusions

In conclusion, we confirmed that the spontaneous genetic mutation T329S in *FMO3* could reduce the development of AS lesions, the incidence of FLS, and fat deposition, which are associated with changes in plasma and hepatic metabolic indices and improvements in the laying performance of 90-week-old layers. Our results suggest an association among T329S, AS lesions, FLS, adiposity, and the laying performance of older layers.

## Data Availability Statement

The original contributions presented in the study are included in the article/Supplementary Material, further inquiries can be directed to the corresponding author/s.

## Ethics Statement

This study was performed in accordance with the Chinese guidelines for animal welfare and experimental protocols. It was approved by the China Agricultural University Animal Experiment Ethics Committee (CAU20160916-2).

## Author Contributions

JS was involved in the conception of the study and the study design, prepared the manuscript, and collected data. XS and XL collected the samples. QL and LZ participated in some experiments. GL and YY provided the animals. GX and JZ were responsible for direction and funding acquisition of the experiment. All authors contributed to the article and approved the submitted version.

## Funding

This study was supported by the National Key Research and Development Program of China (2021YFD1200803); the China Agriculture Research Systems (CARS-41); the Program for Changjiang Scholars and Innovative Research Team in University (IRT_15R62).

## Conflict of Interest

GL and YY was employed by Beijing Huadu Yukou Poultry Industry Co. Ltd. The remaining authors declare that the research was conducted in the absence of any commercial or financial relationships that could be construed as a potential conflict of interest.

## Publisher's Note

All claims expressed in this article are solely those of the authors and do not necessarily represent those of their affiliated organizations, or those of the publisher, the editors and the reviewers. Any product that may be evaluated in this article, or claim that may be made by its manufacturer, is not guaranteed or endorsed by the publisher.

## References

[1] Krueger SK Williams DE. Mammalian flavin-containing monooxygenases: structure/function, genetic polymorphisms and role in drug metabolism. Pharmacol Ther. (2005) 106:357–87. 10.1016/j.pharmthera.2005.01.001 15922018 PMC1828602

[2] Mo F Zheng J Wang P Lian L Yi G Xu G. Quail FMO3 gene cloning, tissue expression profiling, polymorphism detection and association analysis with fishy taint in eggs. PloS ONE. (2013) 8:e81416. 10.1371/journal.pone.0081416 24282592 PMC3840012

[3] Fennema D Phillips IR Shephard EA. Trimethylamine and trimethylamine N-Oxide, a flavin-containing monooxygenase 3 (FMO3)-mediated host-microbiome metabolic axis implicated in health and disease. Drug Metab Dispos. (2016) 44:1839–50. 10.1124/dmd.116.070615 27190056 PMC5074467

[4] Phillips IR Shephard EA. Flavin-containing monooxygenases: mutations, disease and drug response—ScienceDirect. Trends Pharmacol Sci. (2008) 29:294–301. 10.1016/j.tips.2008.03.004 18423897

[5] Honkatukia M Reese K Preisinger R Tuiskula-Haavisto M Weigend S Roito J. Fishy taint in chicken eggs is associated with a substitution within a conserved motif of the FMO3 gene. Genomics. (2005) 86:225–32. 10.1016/j.ygeno.2005.04.005 15916878

[6] Wang J Yue HY Xia ZQ Wu SG Zhang HJ Ji F. Effect of dietary choline supplementation under different flavin-containing monooxygenase 3 genotypes on trimethylamine metabolism in laying hens. Poult Sci. (2012) 91:2221–8. 10.3382/ps.2011-02074 22912456

[7] Wang Z Klipfell E Bennett BJ Koeth R Levison BS Dugar B. Gut flora metabolism of phosphatidylcholine promotes cardiovascular disease. Nature. (2011) 472:57–63. 10.1038/nature09922 21475195 PMC3086762

[8] Zhu W Gregory JC Org E Buffa JA Gupta N Wang Z. Gut microbial metabolite TMAO enhances platelet hyperreactivity and thrombosis risk. Cell. (2016) 165:111–24. 10.1016/j.cell.2016.02.011 26972052 PMC4862743

[9] Seldin MM Meng Y Qi H Zhu W Wang Z Hazen SL. Trimethylamine N-oxide promotes vascular inflammation through signaling of mitogen-activated protein kinase and nuclear factor-kappa B. J Am Heart Assoc. (2016) 5:e002767. 10.1161/JAHA.115.002767 26903003 PMC4802459

[10] Bennett BJ Vallim TQDA Zeneng W Shih DM Yonghong M Jill G. Trimethylamine-N-oxide, a metabolite associated with atherosclerosis, exhibits complex genetic and dietary regulation. Cell Metab. (2013) 17:49–60. 10.1016/j.cmet.2012.12.011 23312283 PMC3771112

[11] Shih DM Wang Z Lee R Meng Y Che N Charugundla S. Flavin containing monooxygenase 3 exerts broad effects on glucose and lipid metabolism and atherosclerosis. J Lipid Res. (2015) 56:22–37. 10.1194/jlr.M051680 25378658 PMC4274068

[12] Warrier M Shih DM Burrows AC Ferguson D Gromovsky AD Brown AL. The TMAO-generating enzyme flavin monooxygenase 3 is a central regulator of cholesterol balance. Cell Rep. (2015) 10:326–38. 10.1016/j.celrep.2014.12.036 25600868 PMC4501903

[13] Shih DM Zhu W Schugar RC Meng Y Jia X Miikeda A. Genetic deficiency of Flavin-containing monooxygenase 3 (FMO3) protects against thrombosis but has only a minor effect on plasma lipid levels—brief report. Arterioscler Thromb Vasc Biol. (2019) 39:1045–54. 10.1161/ATVBAHA.119.312592 31070450 PMC6531332

[14] Tycko J Myer VE Hsu PD. Methods for optimizing CRISPR-Cas9 genome editing specificity. Mol Cell. (2016) 63:355–70. 10.1016/j.molcel.2016.07.004 27494557 PMC4976696

[15] Ward AK Classen HL Buchanan FC. Fishy-egg tainting is recessively inherited when brown-shelled layers are fed canola meal. Poult Sci. (2009) 88:714–21. 10.3382/ps.2008-00430 19276413

[16] Wang J Long C Zhang H Zhang Y Wang H Yue H. Genetic variant in flavin-containing monooxygenase 3 alters lipid metabolism in laying hens in a diet-specific manner. Int J Biol Sci. (2016) 12:1382–93. 10.7150/ijbs.16472 27877090 PMC5118784

[17] Guo YY. Effect of FMO3 gene expression and enzyme activity on duck eggs fish odor. [Dissertation/master's thesis]. China Agricultural University, Beijing, China (2018).

[18] Li H Li Y Yang L Zhang D Liu Z Wang Y. Identification of a novel lipid metabolism-associated hepatic gene family induced by estrogen via ERα in chicken (*Gallus gallus*). Front Genet. (2020) 11:271. 10.3389/fgene.2020.00271 32296460 PMC7136477

[19] Song J Huang M Shi X Li X Chen X He Z. T329S mutation in the FMO3 gene alleviates lipid metabolic diseases in chickens in the late laying period. Animals. (2022) 12:48. 10.3390/ani12010048 35011153 PMC8749748

[20] Harms CFSH. Aortic atherosclerosis in nonlaying hens with fatty liver syndrome. Avian Dis. (1983) 27:652–9. 10.2307/1590307 6639549

[21] Schugar RC Shih DM Warrier M Helsley RN Burrows A Ferguson D. Erratum: the TMAO-producing enzyme flavin-containing monooxygenase 3 regulates obesity and the beiging of white adipose tissue. Cell Rep. (2017) 19:2451–61. 10.1016/j.celrep.2017.05.077 28636934 PMC5672822

[22] Tutunchi H Naeini F Saghafi-Asl M Farrin N Ostadrahimi A. Effects of oleoylethanolamide supplementation on atherogenic indices and hematological parameters in patients with nonalcoholic fatty liver disease: a clinical trial. Health Promot Perspect. (2020) 10:373–82. 10.34172/hpp.2020.56 33312933 PMC7722997

[23] Bain MM Nys Y Dunn IC. Increasing persistency in lay and stabilising egg quality in longer laying cycles. What are the challenges? Br Poult Sci. (2016) 57:330–8. 10.1080/00071668.2016.1161727 26982003 PMC4940894

[24] Aziza AE Awadin W Cherian G. Impact of choline supplementation on hepatic histopathology, phospholipid content, and tocopherol status in layer hens fed flaxseed. J Appl Poult Res. (2019) 28:679–87. 10.3382/japr/pfz019

[25] Walzem RL Hansen RJ Williams DL Hamilton RL. Estrogen induction of VLDLy assembly in egg-laying hens. J Nutr. (1999) 129:467S. 10.1093/jn/129.2.467S 10064311

[26] Tian WH Wang Z Yue YX Li H Li ZJ Han RL. MiR-34a-5p increases hepatic triglycerides and total cholesterol levels by regulating ACSL1 protein expression in laying hens. Int J Mol Sci. (2019) 20:4420. 10.3390/ijms20184420 31500376 PMC6770783

[27] Subcommittee on Poultry Nutrition; Committee on Animal Nutrition; 380 Board on Agriculture; National Research Council. Nutrient Requirements of Poultry. 9th ed. Washington, DC: National Academy Press (1994). p. 155.

[28] Zhang LC. Studies on genetic parameters for and candidate genes associated with egg quality traits in chickens. [Dissertation/doctor's thesis]. China Agricultural University, Beijing, China (2007).

[29] Chen ML Yi L Zhang Y Zhou X Ran L Yang J. Resveratrol attenuates trimethylamine-N-oxide (TMAO)-Induced atherosclerosis by regulating TMAO synthesis and bile acid metabolism via remodeling of the gut microbiota. MBio. (2016) 7:e2210–5. 10.1128/mBio.02210-15 27048804 PMC4817264

[30] Collins HL Drazul-Schrader D Sulpizio AC Koster PD Williamson Y Adelman SJ. L-Carnitine intake and high trimethylamine N-oxide plasma levels correlate with low aortic lesions in ApoE-/- transgenic mice expressing CETP. Atherosclerosis. (2016) 244:29–37. 10.1016/j.atherosclerosis.2015.10.108 26584136

[31] Gao X Liu P Wu C Wang T Liu G Cao H. Effects of fatty liver hemorrhagic syndrome on the AMP-activated protein kinase signaling pathway in laying hens. Poult Sci. (2019) 98:2201–10. 10.3382/ps/pey586 30608557

[32] Lv Z Xing K Li G Liu D Guo Y. Dietary genistein alleviates lipid metabolism disorder and inflammatory response in laying hens with fatty liver syndrome. Front Physiol. (2018) 9:1493. 10.3389/fphys.2018.01493 30405443 PMC6207982

[33] Schneider CA Rasband WS Eliceiri KW. NIH Image to ImageJ: 25 years of image analysis. Nat Methods. (2012) 9:671–5. 10.1038/nmeth.2089 22930834 PMC5554542

[34] Bradford MM. A rapid and sensitive method for the quantitation of microgram quantities of protein utilizing the principle of protein-dye binding. Anal Biochem. (1976) 72:248–54. 10.1016/0003-2697(76)90527-3 942051

[35] Tang WHW Wang Z Levison BS Koeth RA Britt EB Fu X. Intestinal microbial metabolism of phosphatidylcholine and cardiovascular risk. N Engl J Med. (2013) 368:1575–84. 10.1056/NEJMoa1109400 23614584 PMC3701945

[36] Wang Z Roberts AB Buffa JA Levison BS Zhu W Org E. Non-lethal inhibition of gut microbial trimethylamine production for the treatment of atherosclerosis. Cell. (2015) 163:1585–95. 10.1016/j.cell.2015.11.055 26687352 PMC4871610

[37] Koeth RA Wang Z Levison BS Buffa JA Org E Sheehy BT. Intestinal microbiota metabolism of L-carnitine, a nutrient in red meat, promotes atherosclerosis. Nat Med. (2013) 19:576–85. 10.1038/nm.3145 23563705 PMC3650111

[38] Ayala I Pérez BG Doménech G Castells MT Valdés M. Use of the chicken as an experimental animal model in atherosclerosis. Avian Poult Biol Rev. (2005) 16:151–9. 10.3184/147020605783437968

[39] Miao J Ling AV Manthena PV Gearing ME Graham MJ Crooke RM. Flavin-containing monooxygenase 3 as a potential player in diabetes-associated atherosclerosis. Nat Commun. (2015) 6:6498. 10.1038/ncomms7498 25849138 PMC4391288

[40] Marchesini G Bugianesi E Forlani G Cerrelli F Lenzi M Manini R. Nonalcoholic fatty liver, steatohepatitis, and the metabolic syndrome. Hepatology. (2003) 37:917–23. 10.1053/jhep.2003.50161 12668987

[41] Korenblat KM Fabbrini E Mohammed BS Klein S. Liver, muscle, and adipose tissue insulin action is directly related to intrahepatic triglyceride content in obese subjects. Gastroenterology. (2008) 134:1369–75. 10.1053/j.gastro.2008.01.075 18355813 PMC2629391

[42] Laviola L Perrini S Cignarelli A Natalicchio A Leonardini A Stefano FD. Insulin signaling in human visceral and subcutaneous adipose tissue *in vivo*. Diabetes. (2006) 55:952–61. 10.2337/diabetes.55.04.06.db05-1414 16567516

[43] Zhou B Jia L Zhang Z Xiang L Yuan Y Zheng P. The nuclear orphan receptor NR2F6 promotes hepatic steatosis through upregulation of fatty acid transporter CD36. Adv Sci. (2020) 7:2002273. 10.1002/advs.202002273 33173745 PMC7610302

[44] Zhang S Shen YR Wu S Xiao YQ He Q Shi SR. The dietary combination of essential oils and organic acids reduces Salmonella enteritidis in challenged chicks. Poult Sci. (2019) 98:6349–55. 10.3382/ps/pez457 31393588 PMC8913765

[45] Wilkinson MJ Manoogian ENC Zadourian A Lo H Fakhouri S Shoghi A. Ten-hour time-restricted eating reduces weight, blood pressure, and atherogenic lipids in patients with metabolic syndrome. Cell Metab. (2020) 31:92–104. 10.1016/j.cmet.2019.11.004 31813824 PMC6953486

[46] Shini S Stewart GD Shini A Bryden W. Mortality rates and causes of death in laying hens kept in cage and alternative housing systems. In: Proceedings of the 12th European Poultry Conference, Verona, Italy, 10–14 September 2006. Vol. 62. World Poultry Science Association, Beekbergen (2006). p. 601.

[47] Chen Y Liu Y Zhou R Chen X Wang C Tan X. Associations of gut-flora-dependent metabolite trimethylamine-N-oxide, betaine and choline with non-alcoholic fatty liver disease in adults. Sci Rep. (2016) 6:19076. 10.1038/srep19076 26743949 PMC4705470

[48] Tirosh A Shai I Bitzur R Kochba I Tekes-Manova D Israeli E. Changes in triglyceride levels over time and risk of type 2 diabetes in young men. Diabetes Care. (2008) 31:2032–7. 10.2337/dc08-0825 18591400 PMC2551650

[49] Wang L Zhang B Huang F Liu B Xie Y. Curcumin inhibits lipolysis via suppression of ER stress in adipose tissue and prevents hepatic insulin resistance. J Lipid Res. (2016) 57:1243–55. 10.1194/jlr.M067397 27220352 PMC4918853

[50] Perry RJ Camporez JPG Kursawe R Titchenell PM Zhang DY Perry CJ. Hepatic acetyl CoA links adipose tissue inflammation to hepatic insulin resistance and type 2 diabete. Cell. (2015) 160:745–58. 10.1016/j.cell.2015.01.012 25662011 PMC4498261

[51] Nie L Li CP Li JH Chen X Zhong X. Analysis of nonalcoholic fatty liver disease microRNA expression spectra in rat liver tissues. Mol Med Rep. (2018) 18:2669–80. 10.3892/mmr.2018.9268 30015905 PMC6102666

[52] Qiu K Zhao Q Wang J Qi G Wu S Zhang H. Effects of pyrroloquinoline quinone on lipid metabolism and Anti-Oxidative capacity in a high-fat-diet metabolic dysfunction-associated fatty liver disease chick model. Int J Mol Sci. (2021) 22:1458. 10.3390/ijms22031458 33535680 PMC7867196

[53] Shi S Wu S Shen Y Zhang S Xiao Y He X. Iron oxide nanozyme suppresses intracellular salmonella enteritidis growth and alleviates infection *in vivo*. Theranostics. (2018) 8:6149–62. 10.7150/thno.29303 30613289 PMC6299686

[54] Sliz E Sebert S Würtz P Kangas AJ Soininen P Lehtimäki T. NAFLD risk alleles in PNPLA3, TM6SF2, GCKR and LYPLAL1 show divergent metabolic effects. Hum Mol Genet. (2018) 27:2214–23. 10.1093/hmg/ddy124 29648650 PMC5985737

[55] Kim MH Seong JB Huh J Bae YC Lee H Lee D. Peroxiredoxin 5 ameliorates obesity-induced non-alcoholic fatty liver disease through the regulation of oxidative stress and AMP-activated protein kinase signaling. Redox Biol. (2020) 28:101315. 10.1016/j.redox.2019.101315 31505325 PMC6736789

[56] Zhang S Zhong G Shao D Wang Q Hu Y Wu T. Dietary supplementation with bacillus subtilis promotes growth performance of broilers by altering the dominant microbial community. Poult Sci. (2021) 100:100935. 10.1016/j.psj.2020.12.032 33652528 PMC7936199

[57] Pearson AW Butler EJ. Effects of selective breeding and age on the ability of the domestic fowl (gallus domesticus) to oxidize trimethylamine. Comp Biochem Physiol. (1983) 76:67–74. 10.1016/0742-8413(83)90045-2 6139259

