# Supplementary material for: Associations of the T329S Polymorphism in Flavin-Containing Monooxygenase 3 With Atherosclerosis and Fatty Liver Syndrome in 90-Week-Old Hens
Source: Front Vet Sci. 2022 Mar 30;9:868602. doi: 10.3389/fvets.2022.868602 (PMC9009339; doi:10.3389/fvets.2022.868602)
Supplement: Supplementary file 1 [file Table_1.docx]

**Table S1. Composition and nutrient level of the basal diet**

| **Item** | **basal diet** |
| --- | --- |
| *Ingredient (%)* |  |
| Corn | 61.95 |
| Soybean meal | 27.50 |
| Soybean oil | 0.75 |
| Limestone | 8.50 |
| Sodium Salt | 0.30 |
| Premix^1^ | 1.00 |
| Total | 100.00 |
| *Nutrient level^2^* (%) |  |
| ME (MJ/kg) | 11.34 |
| Crude protein | 15.52 |
| Lysine | 0.71 |
| Methionine | 0.36 |
| Threonine | 0.66 |
| Methionine + Cysteine | 0.53 |
| Calcium | 3.40 |
| Available phosphorus | 0.28 |

^1^Premix provided the following per kilogram of diets: Vitamin A, 10000 IU; Vitamin D_3_, 3000 IU; Vitamin E, 30 IU; Vitamin K_3_ 1 mg; Vitamin B_1_, 1 mg; Vitamin B_2_, 6 mg; Vitamin B_6_, 3 mg; Vitamin B_12_, 0.01 mg; biotin, 0.1 mg; folic acid, 0.3 mg; calcium pantothenate, 10 mg; niacin, 45 mg; Cu (as copper sulfate), 8 mg; Fe (as ferrous sulfate), 80 mg; Mn (as manganese sulfate), 100 mg; Zn (as zinc sulfate), 60 mg; I (as potassium iodide), 1 mg; Se (as sodium selenite), 0.3 mg.

^2^Crude protein and calcium levels were analyzed. Each value was based on triplicate determinations, but all other nutrient levels were calculated.
