# Supplementary material for: Associations of the T329S Polymorphism in Flavin-Containing Monooxygenase 3 With Atherosclerosis and Fatty Liver Syndrome in 90-Week-Old Hens
Source: Front Vet Sci. 2022 Mar 30;9:868602. doi: 10.3389/fvets.2022.868602 (PMC9009339; doi:10.3389/fvets.2022.868602)
Supplement: Supplementary file 2 [file Table_2.docx]

**Table S2.** **The categorization scheme of pathological scores of fatty liver**

| **Pathological type** | **Histopathological changes** | **Degree** | **Scores** |
| --- | --- | --- | --- |
| Steatosis | Indicating the normal state. | None | 0 |
|  | The lipid deposition was between 0 and 1 points. | ± | 0.5 |
|  | The fat vacuoles in the liver cells were small and scattered. | Minor lesions + | 1 |
|  | The fat vacuoles in the liver cells are larger and wider in range. | Moderate lesions ++ | 2 |
|  | The fat vacuoles are fused into large vacuoles, and the nucleus is squeezed into the cell membrane, similar to the adipocyte. | Severe lesions +++ | 3 |

The pathological scores of fatty liver are divided into five grades (0–3), according to the levels of steatosis.
